# Chatbot-supported psychoeducation in adult attention-deficit hyperactivity disorder: randomised controlled trial

**DOI:** 10.1192/bjo.2023.573

**Published:** 2023-10-13

**Authors:** Benjamin Selaskowski, Meike Reiland, Marcel Schulze, Behrem Aslan, Kyra Kannen, Annika Wiebe, Torben Wallbaum, Susanne Boll, Silke Lux, Alexandra Philipsen, Niclas Braun

**Affiliations:** Department of Psychiatry and Psychotherapy, University Hospital Bonn, Germany; Department of Information and Communication, Flensburg University of Applied Sciences, Germany; Department of Computing Science, University of Oldenburg, Germany

**Keywords:** Attention-deficit hyperactivity disorder, chatbot, smartphone-assisted psychoeducation, conversational agent, digital health

## Abstract

**Background:**

Although psychoeducation is generally recommended for the treatment of adult attention-deficit hyperactivity disorder (ADHD), participation in clinical psychoeducation groups is impeded by waiting times and the constrained number of patients who can simultaneously attend a group. Digital psychoeducation attempts are promising, but the rapidly expanding number of apps lack evidence and are mostly limited to only a few implemented interactive elements.

**Aims:**

To determine the potential of digital, self-guided psychoeducation for adult ADHD, a newly developed interactive chatbot was compared with a previously validated, conventional psychoeducation app.

**Method:**

Forty adults with ADHD were randomised, of whom 17 participants in each group completed self-guided psychoeducation based on either a chatbot or conventional psychoeducation app between October 2020 and July 2021. ADHD core symptoms were assessed before and after the 3-week interventions, using both the blinded observer-rated Integrated Diagnosis of ADHD in Adulthood interview and the self-rated ADHD Self-Assessment Scale (ADHS-SB).

**Results:**

Observer- and patient-rated ADHD symptoms were significantly reduced from pre- to post-intervention (observer-rated: mean difference −6.18, 95% CI −8.06 to −4.29; patient-rated: mean difference −2.82, 95% CI −4.98 to −0.67). However, there were no group × intervention interaction effects that would indicate a stronger therapeutic benefit of one of the interventions. Likewise, administered psychoeducational knowledge quizzes did not show differences between the groups. No adverse events were reported.

**Conclusions:**

Self-guided psychoeducation based on a chatbot or a conventional app appears similarly effective and safe for improving ADHD core symptoms. Future research should compare additional control interventions and examine patient-related outcomes and usability preferences in detail.

With a prevalence of approximately 5.9% in youth and 2.5% in adulthood,^1,2^ attention-deficit hyperactivity disorder (ADHD) is a neurodevelopmental disorder associated with substantial individual suffering and economic burden.^3,4^ Clinical complexity is further aggravated by high rates of comorbid disorders, such as substance use disorder, depression, bipolar disorder and anxiety disorders.^5^ Pharmacological treatment has effectively reduced ADHD symptoms and is considered the first-line treatment.^6–8^ Yet, it is associated with side-effects,^9^ risks of multimorbid pharmacotherapy and issues with adherence.^10^ In addition, the non-medical use of prescribed stimulants has emerged as a major public health concern.^11^ Cognitive–behavioural therapy (CBT) is recommended in cases of low pharmacological treatment benefit or to specifically address functional impairment.^6^ Regardless of conducting other treatments, guidelines generally recommend comprehensive psychoeducation.^7^

The basic principles of psychoeducation are to provide knowledge about the disorder and treatment procedures, as well as emphasise the patient's personal strengths and potential for growth. Although psychoeducation generally shows promising results, few rigorous examinations of treatment effects on ADHD core symptoms exist. One of these studies compared psychoeducation with mindfulness training in adults with ADHD,^12^ and another study assessed psychoeducation against CBT in medicated but still symptomatic adults.^13^ Both studies found all interventions to be similarly effective in improving ADHD core symptoms. Compared with other treatments, psychoeducation has the advantage of having hardly any side-effects as well as being easily scalable through digital provision without significantly increasing costs. A psychoeducation mobile app, for instance, could significantly reduce the time and effort associated with conducting clinical psychoeducation.

## Current state of digital health applications

In general, advances in digital health have led to the development of a substantial number of mobile health (mHealth) apps, which can reduce the need for in-person meetings with a clinician, shorten waiting list times, promote self-care^14^ and be economically beneficial because of their low-cost scalability, especially in low-income countries.^15^ However, although mHealth is growing in popularity, scientific evidence of its efficacy is inconsistent, study quality is often low^16^ and, moreover, safety concerns related to incorrect information, lack of expert involvement and poor validation have been reported.^17^ Consequently, the risk of low-value care – that is, services that provide little benefit to patients or that even cause harm – is particularly high.^18^ This risk also applies to mental mHealth, where many applications seem to have no scientifically valid foundation,^19^ and can appear to suggest evidence-based treatments by using misleading scientific language.^20^ Regarding ADHD, for instance, Păsărelu et al.^21^ identified 109 apps, including 23 that focused on psychoeducation, but none provided proof of their effectiveness.

## Digital psychoeducation in adult ADHD

To address this issue, our research group recently conducted a randomised controlled trial (RCT), in which we evaluated an 8-week psychoeducation group programme that was either assisted by traditional paper brochures or by a newly developed psychoeducation app for adult ADHD.^22^ Although the app was more effective in improving ADHD symptoms and no adverse events were reported, we cannot directly transfer these results to self-guided psychoeducation without a concomitant psychoeducation group. Moreover, the app used may be less suitable for self-guided learning in ADHD because it was developed as a digital, but hardly interactive, instructional format. Thus, the potential benefits of mHealth applications, such as considering individual patient differences in learning behaviour, have not yet been incorporated.

Considering that motivation-related and dysfunctional learning behaviours occur in adult ADHD, the implementation of a psychoeducation chatbot may be of particular value. A chatbot (i.e. a conversational agent) is a computer program that can simulate conversations with human users. Potential benefits for psychoeducation include the possibility of interactively self-guiding the learning path, and receiving individualised responses and feedback that are not achievable with a ‘conventional’ psychoeducation app. Although these properties appear valuable for several mental disorders, there is limited evidence for the use of chatbots in mental health.^23^ Regarding attention deficits, although not specifically addressing patients with ADHD, only one previous study conducted a chatbot-assisted psychoeducation, and found stronger improvements of ADHD-related symptoms than a self-help book control group.^24^

## Aims

In this study, we implemented a new chatbot that, based on validated psychoeducational content, interacts in such a way that the patient co-determines the topics addressed. We hypothesised that this approach might lead to greater symptom improvement than conventional module-based content presentation, given the increased potential for self-guidance through the preferred psychoeducational content, as well as the higher level of interaction and individualisation offered by a chatbot. For clinical evaluation, we conducted an RCT to evaluate the effects of a 3-week self-guided, chatbot-based psychoeducation (CBP) in adults with ADHD compared with our previously validated psychoeducation app, which is based on a module-by-module content presentation.^22^

## Method

### Participants

A total of 139 adult out-patients with ADHD were contacted for study participation, of which 40 participants were randomised to the intervention groups and 34 participants completed the study (for the participant flow chart, see Supplementary Fig. 1 available at https://doi.org/10.1192/bjo.2023.573). The study was advertised via the Department of Psychiatry and Psychotherapy of the University Hospital Bonn, via the website ‘Central ADHD Network’ (https://www.zentrales-adhs-netz.de), and via other publicly accessible media. Individuals were eligible to participate if they met DSM-5 ADHD diagnostic criteria,^25^ as assessed by the observer-rated Integrated Diagnosis of ADHD in Adulthood (IDA-R);^26^ were aged 18–65 years; had access to a smartphone with Android OS (version 5.0 or higher) and had sufficient command of the German language.

Individuals were ineligible to participate if they met the diagnostic criteria for schizophrenia or other psychotic disorders; severe affective disorder; moderate-to-severe substance use disorder, as assessed by the Brief Diagnostic Interview for Mental Disorders (Mini-Dips-OA, German version);^27^ or antisocial personality disorder, as evaluated by the Assessment of DSM-IV Personality Disorders (ADP-IV, German version).^28^ Intake of medication for ADHD was permitted, but had to be stable from 4 weeks preceding the start of study through to the final examinations. The participants received no compensation for their participation in the study.

The authors assert that all procedures contributing to this work comply with the ethical standards of the relevant national and institutional committees on human experimentation and with the Helsinki Declaration of 1975, as revised in 2008. All procedures involving human patients were approved by the local medical ethics committee of the University of Bonn (protocol number: 123/19). All participants provided written informed consent. The study was preregistered in the German Clinical Trials Register (DRKS; identifier: DRKS00022287) on 13 August 2020. An *a priori* sample calculation in G*Power version 3.1 for Windows^29^ (Faul F, Erdfelder E, Buchner A and Lang A-G, Heinrich Heine University, Düsseldorf, Germany; see https://www.psychologie.hhu.de/arbeitsgruppen/allgemeine-psychologie-und-arbeitspsychologie/gpower) was performed to determine the required sample size of 34 participants, which was based on an alpha error probability of 0.05, a power of 80% and a moderate effect size (*f* = 0.25). To account for study drop-out, a randomisation of *n* = 40 was pursued. The first participant was enrolled on 29 September 2020.

### Study design

Two interventions were compared in this parallel-group RCT: a self-guided CBP and a self-guided, app-based psychoeducation (ABP). All participants underwent an extensive baseline assessment (time point 0) and were allocated to one of the intervention groups by permuted block randomisation in blocks of two, to maintain balanced group sizes. Sequence generation and participant enrolment were performed by different study personnel. Participants were asked to engage with the psychoeducation content as much as possible during the self-guided 3-week psychoeducation period. Afterwards, a final assessment (time point 1) was conducted. The relative change in ADHD total symptom severity from time point 0 to time point 1, as examined by a blinded clinical rater on the IDA-R, was considered the primary outcome parameter of the study. Participants’ remarks about app specifics during the final assessment (time point 1), which provided indications of their respective assigned group, were the cause for not maintaining rater blinding for five cases in the CBP group and four cases in the ABP group. Participants were not blinded for assignment to CBP or ABP.

### Clinical outcome assessment

Besides observer-rated ADHD symptoms as measured via the IDA-R,^26^ self-rated ADHD symptoms were obtained via the ADHD Self-Assessment Scale (ADHS-SB).^30^ Further outcome parameters included the subscales of the World Health Organization Quality of Life questionnaire (WHOQOL)^31^ and the Depression, Anxiety and Stress Scale (DASS-21).^32^ The Multiple-choice Word Test (MWT-B) was conducted at time point 0 exclusively, to estimate verbal intelligence.^33^

### Procedures

Following baseline assessments at time point 0 (approximately 2 h), participants received a download link to either the conventional psychoeducation app or the chatbot, and time point 1 final assessments were scheduled. However, because of organisational constraints of some participants, these could not be exactly planned after 3 weeks in all cases. As a result, there were marginal differences in the duration of possible use between participants (see ‘Results’). Both the CBP and ABP groups were not limited in the amount or duration of material usage during the intervention period. Processing of all psychoeducational content was estimated to require about 16 h. Following the self-guided 3-week intervention periods, final assessments (approximately 1 h) of the outcome parameters were conducted.

### Interventions

**Fig. 1:**
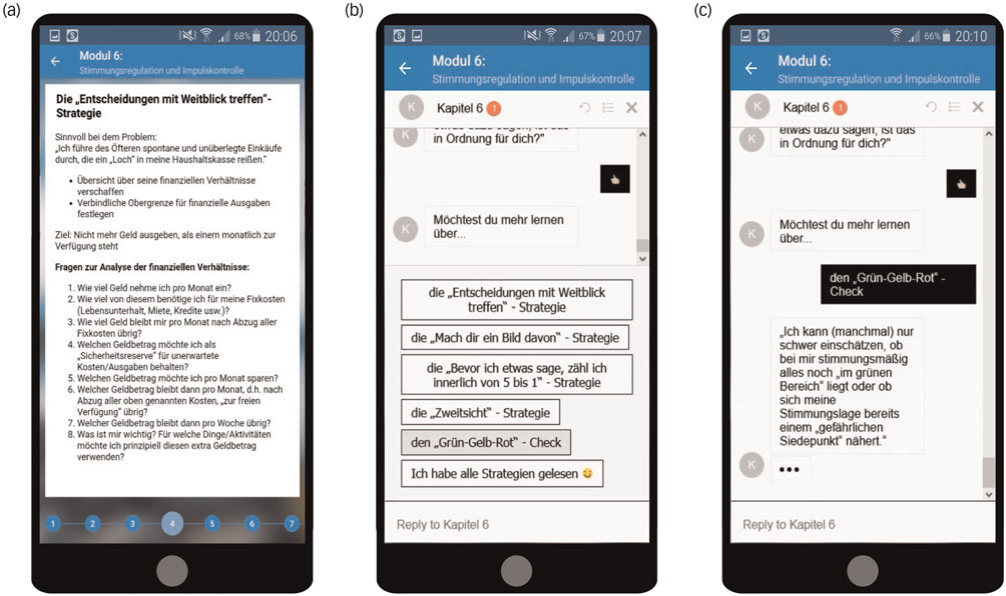
Functionality of the two psychoeducation systems. (a) Presentation of a slide from the emotion regulation module used in the app-based psychoeducation group. The content is presented linearly within each module. The Android app ‘AwareMe ADHS’ was evaluated in a previous study.^22^ (b) Illustration of the chatbot used in the chatbot-based psychoeducation group. Here, participants engaged in ‘digital conversations’ within each module, interacting mainly based on predefined response options, as shown in the bottom section. (c) After selecting an answer, the chatbot responded and presented psychoeducational content or asked additional questions to further narrow down the participant's preferred content.

The psychoeducation content of both interventions was based on a validated manual,^34^ and consisted of eight separate modules that contained a comprehensive summary of the psychoeducation content, assignments and a content quiz. In line with a recent Delphi consensus study on digital psychoeducation for adult ADHD,^35^ a comprehensive summary on various aspects of ADHD was implemented, including multiple illustrations to facilitate understanding. The following topics were addressed: basic information about ADHD, personal resources, mindfulness and attention control, self-organisation, stress management, mood regulation and impulsive behaviour control, relationships and a final evaluation. Within each module, the conventional psychoeducation app presented the content linearly (i.e. module by module), whereas the chatbot interactively presented content based on user input (see Fig. 1). The chatbot also offered to present all the information of each module, but the user could skip the content more easily compared with the conventional app. Both the chatbot and the conventional app included identical quiz questions at the end of each module (except for evaluation module eight), to evaluate the acquired psychoeducational knowledge.

The conventional psychoeducation app of the ABP group is available in the Google Play Store (‘AwareMe ADHS’; https://play.google.com/store/apps/details?id=de.awareme.pse) and has previously been presented in detail.^22^ The chatbot (CBP group) was based on the open-source conversational artificial intelligence platform Botpress version 12.2 for Windows (Botpress, Quebec, Canada; see https://botpress.com).

### Statistical analyses

Full IDA-R and questionnaire data were obtained from all 34 participants who completed the study. In the CBP group, however, 14 of the 17 participants were provided only partial access to module seven of the psychoeducation programme, because of technical errors. Module seven data for both groups was therefore dismissed from analyses.

Separate two × two mixed analyses of variance (see Supplementary Table 1) with group (CBP, ABP) as a between-subjects factor and time (time point 0, time point 1) as a within-subjects factor, were conducted for the following outcome parameters: IDA-R total, inattention (sum score of E1 items), hyperactivity (sum score of E2.1–E2.5 items) and impulsivity (sum score of E2.6–E2.9 items) scores; ADHS-SB total, inattention, hyperactivity and impulsivity scores; the sum scores of the DASS-21 subscales (depression, anxiety and stress) and the sum scores of the WHOQOL subscales (physical health, psychological health, social relationships and environment).

Data from the DASS-21 scales for symptoms of depression and anxiety were considerably right-skewed, resulting in non-normal distributions for scores at both time points (Shapiro–Wilk test, *P* < 0.05). Therefore, the DASS-21 variables of these two scales were Johnson-transformed^36^ and subsequent tests for normal distributions revealed no violations (Shapiro–Wilk test, *P*_T0_ = 0.33, *P*_T1_ = 0.18). Transformed data were used for all statistical analyses.

The percentage of correct responses and the percentage of missing responses of the quiz were compared by using separate independent *t*-tests between both groups. Moreover, an exploratory correlation analysis was conducted between primary and secondary outcome parameters, using time point 0 to time point 1 difference scores. Pearson correlations between each difference score were calculated separately for each intervention group.

Statistical analyses were conducted with SPSS software version 21.0 for Windows^37^ and MATLAB version 2021b for Windows (The MathWorks, Massachusetts, USA; see https://de.mathworks.com/products/matlab.html). Visualisation of the correlation matrix was performed with R version 3.6.1 for Windows (R Core Team, Vienna, Austria; see https://www.R-project.org),^38^ using the Corrplot package for R version 0.84.^39^ Reported statistical tests were two-sided and based on a significance level of *α* = 0.05.

## Results

### Sample characterisation and demographics

In total, 34 participants (18 women, 16 men; mean age 29.6 years, s.d. 8.4) completed the RCT between October 2020 and July 2021. Table 1 provides a presentation of the balanced clinical baseline and demographic characteristics for the CBP (*n* = 17) and ABP (*n* = 17) groups. The exact duration from time point 0 to time point 1 in which the participants could access the psychoeducation content on their smartphones was 22.5 days (s.d. 2.2) in the CBP group and 23.8 days (s.d. 4.3) in the ABP group (*t*(32) = 1.16; *P* = 0.25).

**Table 1:** Demographic and clinical sample characteristics

	*N* (%)		*P*-value^a^
Characteristic	CBP (*n* = 17)	ABP (*n* = 17)	Group comparison
Age, years			
Mean (s.d.)	29.6 (7.6)	29.7 (9.5)	0.99
Range	19–44	20–52	
Female	9 (52.9%)	9 (52.9%)	0.92
University entrance diploma			
(year 5 to 12/13)	12 (70.6%)	16 (94.1%)	0.72
Full- or part-time employment	8 (47.1%)	11 (64.7%)	0.30
Verbal IQ, mean (s.d.)	106.5 (13.1)	109.7 (14.0)	0.48
Previous psychoeducation experience	4 (23.5%)	6 (37.5%)	0.38
ADHD presentation			0.30
Inattentive	2 (11.8%)	3 (17.7%)	
Hyperactive–impulsive	0	1 (5.9%)	
Combined	15 (88.2%)	13 (76.5%)	
Psychopharmacological treatments			
Methylphenidate	10 (58.8%)	5 (29.4%)	0.08
Amphetamine	0	3 (17.6%)	0.70
Other psychostimulants	0	0	
Atomoxetine	0	1 (5.9%)	0.31
Antidepressant	2 (11.8%)	6 (35.3%)	0.11
Anticonvulsants, anxiolytics, antipsychotics, others	3 (17.6%)	2 (11.8%)	0.63
No medication	3 (17.6%)	4 (23.5%)	0.67
Comorbid mental disorders			
Affective disorders	8 (47.1%)	11 (64.7%)	0.30
Anxiety disorders	5 (29.4%)	8 (47.1%)	0.29
Comorbid personality disorders			
Schizoid, schizotypal, paranoid	1 (5.9%)	1 (5.9%)	0.38
Borderline, narcissistic, histrionic	4 (23.5%)	1 (5.9%)	0.15
Avoidant, obsessive–compulsive, dependent	4 (23.5%)	3 (17.6%)	0.67

CBP, chatbot-based psychoeducation; ABP, app-based psychoeducation; ADHD, attention-deficit hyperactivity disorder.

a. Based on independent *t*-tests or chi-squared tests.

### ADHD symptom severity

**Fig. 2:**
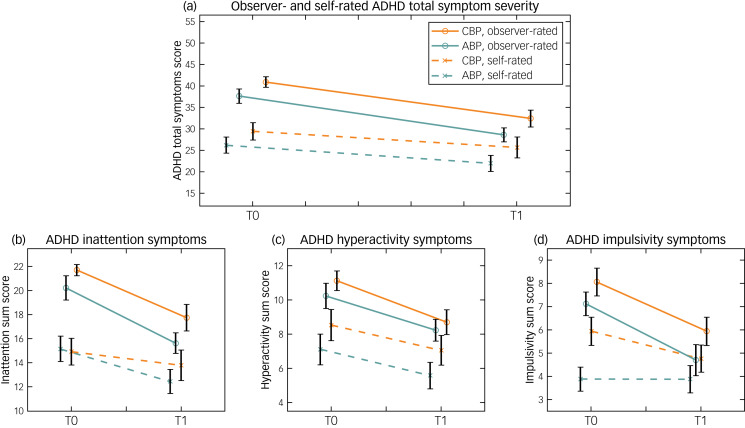
Observer- and self-rated ADHD symptom severity before (time point 0) and after (time point 1) the 3-week psychoeducation interventions. The (a) ADHD total symptom scores and subscores for symptoms of (b) inattention, (c) hyperactivity and (d) impulsivity based on IDA-R observer ratings (solid line) and ADHS-SB self-ratings (dashed line) are presented. The chatbot-based psychoeducation group (orange line) and the app-based psychoeducation group (green line) are depicted separately. The IDA-R and ADHS-SB total scores ranged from 0 to 54. The maximum values for inattention, hyperactivity and impulsivity scores were 27, 15 and 12, respectively. Error bars indicate standard errors of the mean. ABP, app-based psychoeducation; ADHD, attention-deficit hyperactivity disorder; ADHS-SB, ADHD Self-Assessment Scale; CBP, chatbot-based psychoeducation; IDA-R, Integrated Diagnosis of ADHD in Adulthood; T0, time point 0; T1, time point 1.

Changes in observer- and self-rated ADHD symptoms from time point 0 to time point 1 are shown in Fig. 2. The analysis of observer-rated ADHD symptoms showed that IDA-R total scores decreased from time point 0 to time point 1 (mean difference −6.18, 95% CI −8.06 to −4.29) across groups (F(1,32) = 44.44; *P* < 0.001; ηp^2^ = 0.58), with reductions of 20.8% in the CBP group and 23.9% in the ABP group. Neither a group × time interaction (F(1,32) = 0.04; *P* = 0.84), nor a significant main effect of group (F(1,32) = 3.47; *P* = 0.072) was found.

In line with this, the separate analyses of each core symptom (i.e. IDA-R subscale scores) showed no significant group × time interactions for inattention (F(1,32) = 0.17; *P* = 0.68), hyperactivity (F(1,32) = 0.16; *P* = 0.69) or impulsivity (F(1,32) = 0.15; *P* = 0.70), but only showed main effects of time. That is, across groups, inattention improved by 20.3% (F(1,32) = 30.30; *P* < 0.001; ηp² = 0.47), hyperactivity improved by 20.7% (F(1,32) = 18.30; *P* < 0.001; ηp² = 0.36) and impulsivity improved by 29.9% (F(1,32) = 34.90; *P* < 0.001; ηp² = 0.52) from time point 0 to time point 1. No main effect of group on any core symptom was revealed.

Self-rated ADHD total symptoms (i.e. ADHS-SB total score) also improved (mean difference −2.82, 95% CI −4.98 to −0.67) over time (F(1,32) = 7.12; *P* = 0.012; ηp² = 0.18), but no group × time interaction (F(1,32) = 0.03; *P* = 0.88) was observed. Here, ADHD total symptoms decreased by 12.6% in the CBP group and 16.4% in the ABP group, with no significant main effect of group on total symptom severity (F(1,32) = 1.93; *P* = 0.17).

In the separate analyses of self-rated ADHD core symptoms (i.e. ADHS-SB subscale scores), no group × time interactions were found, but time had a significant main effect. Specifically, we observed improvements of 12.7% for inattention (F(1,32) = 6.77; *P* = 0.014; ηp² = 0.18) and 19.2% for hyperactivity (F(1,32) = 7.64; *P* = 0.009; ηp² = 0.19), but only a descriptive reduction of 12.0% for impulsivity (F(1,32) = 1.65; *P* = 0.21; ηp² = 0.05). For impulsivity, in turn, a significant main effect of group (F(1,32) = 4.84; *P* = 0.035; ηp² = 0.13) was found, in that mean impulsivity symptoms were higher in the CBP group (mean 5.35, 95% CI 4.39 to 6.31) than in the ABP group (mean 3.88, 95% CI 2.91 to 4.85).

### Psychoeducational knowledge quiz

The percentages of correct and missing answers in the content quizzes are presented in Supplementary Fig. 2. Of the questions, 21.1% were not answered in the CBP group and 6.9% were not answered in the ABP group. This difference in the percentage of missing responses was, however, not statistically significant (*t*(22,5) = −1.85, *P* = 0.078, *d* = −0.78). Both groups also performed similarly on the content quiz (*t*(32) = 0.62, *P* = 0.54, *d* = 0.22), as measured by the proportion of correct answers, which amounted to 76.4% in the CBP group and 79.4% in the ABP group.

### Symptoms of depression, anxiety and stress

The analyses of comorbid symptoms (i.e. DASS-21 scales) did not reveal any group × time interactions with regard to symptoms of depression (F(1,32) = 2.47; *P* = 0.13), anxiety (F(1,32) = 0.72; *P* = 0.40) or stress (F(1,32) = 0.01; *P* = 0.94). Besides a significant group effect indicating higher stress symptoms in the CBP group (F(1,32) = 5.79; *P* = 0.02; ηp² = 0.15), we did not observe significant main effects of time or group (see Supplementary Table 1).

### Quality of life

The domain-specific analysis of self-rated quality of life (i.e. WHOQOL scales) demonstrated no significant group × time interactions for physical health (F(1,32) < 0.01; *P* = 0.95), psychological health (F(1,32) = 0.47; *P* = 0.50), social relationships (F(1,32) = 0.36; *P* = 0.55) or environment (F(1,32) = 0.12; *P* = 0.73). Instead, the analysis of variance only revealed a significant main effect of group on quality of life concerning social relationships (F(1,32) = 4.27; *P* = 0.047; ηp² = 0.12). Concretely, the ABP group (mean 73.53, 95% CI 65.85 to 81.21) reported higher quality of life than the CBP group (mean 62.50, 95% CI 54.82 to 70.18).

### Correlation analysis

**Fig. 3:**
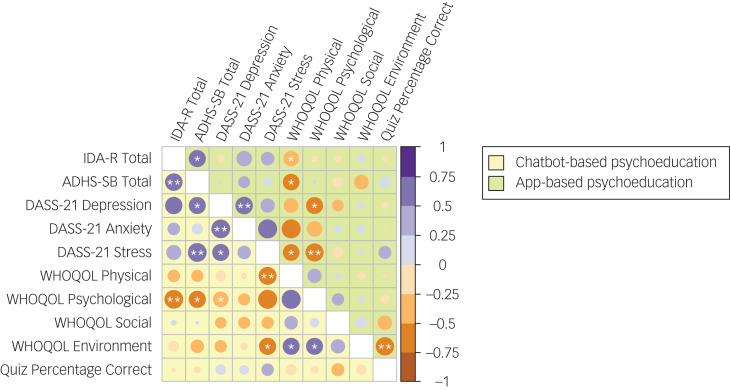
Correlation matrix of study outcome parameters. Pearson correlations (*r*) are depicted separately for the chatbot-based psychoeducation group (below the diagonal, in yellow) and the app-based psychoeducation group (above the diagonal, in green). Correlations between difference scores of observer-rated (IDA-R total score) and self-rated (ADHS-SB total score) ADHD symptoms; symptoms of depression, anxiety and stress (separate DASS-21 subscale scores); quality of life (separate WHOQOL subscale scores for physical health, psychological health, social relationships and environment) and psychoeducational content knowledge (percentage of correct quiz responses) are presented. Except for percentages of correct quiz responses, analyses were based on difference scores from time point 0 to time point 1. ADHD, attention-deficit hyperactivity disorder; ADHS-SB, ADHD Self-Assessment Scale; DASS-21, Depression, Anxiety and Stress Scale; IDA-R, Integrated Diagnosis of ADHD in Adulthood; WHOQOL, World Health Organization Quality of Life questionnaire. *P < 0.05, **P < 0.01.

A detailed matrix of Pearson correlations between time point 0 to time point 1 difference scores of primary and secondary outcome parameters is depicted in Fig. 3. In both groups, positive correlations were found between changes in observer- and self-rated ADHD symptom severities (*r*_CBP_ = 0.64, *P*_CBP_ < 0.01; *r*_ABP_ = 0.59, *P*_ABP_  < 0.05), and between changes in DASS-21 scores for symptoms of depression and anxiety (*r*_CBP_ = 0.70, *P*_CBP_ < 0.01; *r*_ABP_ = 0.62, *P*_ABP_ < 0.01). In the CBP group, *inter alia*, DASS-21 difference scores for depression symptoms were further positively correlated with those of stress (*r* = 0.56, *P* < 0.05), and larger difference scores of stress (*r* = 0.70, *P* < 0.01) and depression (*r* = 0.56, *P* < 0.05) were associated with larger difference scores of self-rated ADHD symptoms.

### Study drop-outs and adverse events

In each group, three participants did not complete the study for unknown reasons, as a reestablishment of contact was unsuccessful. However, no unintended consequences or adverse events related to any intervention were reported.

## Discussion

In this RCT, we examined the efficacy of self-guided digital psychoeducation for adults with ADHD. Specifically, we compared a newly developed chatbot with a previously validated psychoeducation app in addressing ADHD symptoms in a 3-week psychoeducation. A total of 34 participants completed the study and, although both interventions yielded strong effects in the reduction of ADHD core symptoms, neither proved superior.

### Effects of digital psychoeducation on ADHD core symptoms

The symptom improvements found across intervention groups were evident in both observer ratings (approximately 22%) and patient ratings (approximately 15%), with underestimates of self-rated effects being a common finding in adult ADHD, according to a previous meta-analysis.^40^ However, although these results appear promising, it has to be considered that the two interventions in this study were not compared with further control conditions (e.g. a waiting list group). Therefore, we cannot completely rule out potential improvements caused by incidental treatment effects independent of our specific interventions.

Notably, in the current study, we find similar effect sizes in terms of ADHD symptom reductions as in our previous app study.^22^ However, although we previously assessed our conventional psychoeducation app in combination with an 8-week psychoeducation group, in the current study, we tested the app and the chatbot as self-directed psychoeducation approaches without face-to-face meetings or involvement of clinical experts in the treatment process. Therefore, although we earlier demonstrated the effectiveness of a psychoeducation app as an adjunct to a group intervention, here, we provide first evidence that ABP or CBP for adults with ADHD may also be effective in a self-guided setting without continuous clinical supervision.

Regarding symptom-specific effects, our analyses revealed significant and strong effects on observer-rated inattention, hyperactivity and impulsivity, as well as self-rated inattention and hyperactivity across intervention groups. These results contrast with the only other study that examined a psychoeducation chatbot in individuals with attention deficits, which primarily found significant improvements in impulsivity symptoms compared with reading a self-help book, but no improvements in inattention and hyperactivity in their per-protocol analysis.^24^ However, their generalisability may be limited given that they did not include patients with ADHD and only included individuals with attention deficits regardless of psychiatric diagnosis. Still, a recent pre–post feasibility study of a conventional mHealth app for CBT psychoeducation found that adults with ADHD viewed the content delivered via an app positively, and self-reported a decrease in ADHD symptoms after 7 weeks of use.^41^ Also, our results are generally consistent with other psychoeducation studies in adult ADHD, in which all core symptoms improved after the intervention.^12,13^

### Psychoeducational knowledge and secondary outcome evaluation

The evaluation of psychoeducational knowledge transfer showed similar results in terms of knowledge acquisition and content completion in both groups. This is contrary to our expectation, as we hypothesised that a greater emphasis on interaction and the individual selection of topics associated with the use of the chatbot would lead to a greater increase in psychoeducation content knowledge compared with the use of the conventional app. Yet, it is also conceivable that greater involvement and constant interaction may have instead led to a decreased learning capacity in patients with ADHD that overshadowed potential positive effects of individual learning pathways. Future psychoeducation research should additionally focus on the patients’ specific learning styles.

Further secondary outcomes included changes in depression, anxiety, stress and quality of life. In line with our previous study,^22^ which yielded improvements in ADHD core symptoms, but not in secondary outcomes (e.g. depression and functional impairments), we found no enhancement in any secondary outcome in the present study. Although we used narrow inclusion criteria for affective disorders in both our studies and therefore did not expect great reductions in depressive symptoms, improvement in quality of life was particularly anticipated based on previous, non-digital psychoeducation in ADHD.^12,13^ One explanation for this difference could be that in these studies, quality of life was considered as health-related rather than global, as was done in this study by using the WHOQOL. In addition, our correlation analysis found that symptoms of ADHD and symptoms of depression and stress, as well as subdomains of quality of life, generally correlate well with each other. In particular, the health-related subdomains of the WHOQOL (i.e. physical and psychological health) were correlated with changes in other symptom scores. Hence, despite not finding time effects across groups, improvements in ADHD symptoms may be associated with improvements in other secondary domains. As this has relevance for clinical application, future research should target this issue and investigate the extent to which there are potential deviations from non-digital psychoeducation.

In general, both groups reported no adverse events or unintended consequences and had equal drop-out rates. As mentioned above, the chatbot was more prone to technical errors. Consequently, in this study, the conventional psychoeducation app appeared to have advantages in terms of overall usability. Given the similar clinical efficacy, this may also illustrate the potential therapeutic benefit that a more individualised approach, such as a chatbot, could have if the technical foundation allows for a flawless and natural flow of conversation. On the other hand, there is the possibility that the preferred method of content delivery, and ultimately the clinical efficacy, also depends on individual patient characteristics. For example, patients who have no prior experience with psychoeducation may prefer an app that offers a more structured format, whereas more experienced patients may have specific questions and interests that can be addressed more efficiently with a chatbot.

### Limitations and future directions

This study has some limitations. First, the total duration of interventions was rather short and could therefore be responsible for the lack of improvements in secondary outcomes. In particular, changes in quality of life could possibly be perceived in a delayed manner. Second, for technical reasons, we could not limit the duration of app use to precisely 3 weeks. As a result, the average duration of app access was approximately 1 day longer in the ABP group than the CBP group. We assume only minor implications, as completing the amount of psychoeducational content was manageable within the intervention period. Moreover, we were technically unable to measure the exact amount of time each participant spent using the app. Future research should incorporate these measurements, as they may provide important insights into the behaviour of patients with ADHD. Third, although the conventional app worked without errors, the chatbot had some technical issues in one of the modules that eventually led to the exclusion of this module from the analysis and may have resulted in fewer symptom improvements, as well as negative associations with CBP among affected participants. Chatbots appear particularly susceptible in this regard, and conducting pilots with a wide range of devices is recommended. Fourth, the chatbot was compared with an active control intervention that had only been validated once, along with group psychoeducation. Further evaluation of the chatbot against a traditional group psychoeducation or treatment as usual is recommended. Finally, the relatively moderate sample size may have contributed to the failure to detect certain effects, which also complicates more detailed subgroup analysis. However, with respect to the primary end point, we assume that none of the interventions proved superior, given that effect sizes were considerably small.

Overall, taking into account the similar outcome of the digital psychoeducation forms examined in this study, a chatbot may offer the greater potential for development, especially considering the current pace of innovation in the non-clinical chatbot market. Future research should also focus on the implementation of different add-ons that might help patients organise their personal digital psychoeducation, such as notifications that provide exercise reminders and recap psychoeducational content to help deepen knowledge.^14^ In addition, specific participant characteristics that might affect treatment outcomes should be examined, as, for instance, different age groups might have different preferences regarding usability and content presentation, or education level might be related to certain preferred learning styles. In addition, the generalisability of the present results may be limited by the above-average educational level of the sample, which should be addressed in future studies.

In conclusion, we found both a conventional module-based psychoeducation app and an interactive chatbot to be safe, feasible and effective for self-guided psychoeducation, although neither can be favoured based on the present findings. Strong effects on ADHD core symptoms were observed, providing a first step toward implementing these scalable and cost-effective applications in clinical practice; for instance, to provide treatment during therapy waiting times or as an augmentation to medication. Secondary outcome effects, such as on symptoms of depression, anxiety and stress and quality of life, need to be given stronger consideration in the further development of digital psychoeducation, as they did not improve in this study.

## Supporting information

Selaskowski et al. supplementary material Selaskowski et al. supplementary material

## Data Availability

The data that support the findings of this study are available on request from the corresponding author, B.S. Sharing with third parties requires approval.

## References

[1] Faraone SV, Banaschewski T, Coghill D, Zheng Y, Biederman J, Bellgrove MA, et al. The World Federation of ADHD international consensus statement: 208 evidence-based conclusions about the disorder. Neurosci Biobehav Rev 2021; 128: 789–818. 33549739 10.1016/j.neubiorev.2021.01.022 PMC8328933

[2] Song P, Zha M, Yang Q, Zhang Y, Li X, Rudan I. The prevalence of adult attention-deficit hyperactivity disorder: a global systematic review and meta-analysis. J Glob Health 2021; 11: 4009. 10.7189/jogh.11.04009 PMC7916320 33692893

[3] Sciberras E, Streatfeild J, Ceccato T, Pezzullo L, Scott JG, Middeldorp CM, et al. Social and economic costs of attention-deficit/hyperactivity disorder across the lifespan. J Atten Disord 2022; 26(1): 72–87. 33047627 10.1177/1087054720961828

[4] Libutzki B, May M, Gleitz M, Karus M, Neukirch B, Hartman CA, et al. Disease burden and direct medical costs of incident adult ADHD: a retrospective longitudinal analysis based on German statutory health insurance claims data. Eur Psychiatry 2020; 63(1): e86. 32998793 10.1192/j.eurpsy.2020.84 PMC7576526

[5] Chen Q, Hartman CA, Haavik J, Harro J, Klungsøyr K, Hegvik T-A, et al. Common psychiatric and metabolic comorbidity of adult attention-deficit/hyperactivity disorder: a population-based cross-sectional study. PLoS One 2018; 13(9): e0204516. 30256837 10.1371/journal.pone.0204516 PMC6157884

[6] National Institute for Health and Care Excellence (NICE). Attention Deficit Hyperactivity Disorder: Diagnosis and Management. NICE Guideline [NG187]. NICE, 2019 (https://www.nice.org.uk/guidance/ng87). 29634174

[7] German Association of the Scientific Medical Societies (AWMF). *Long Version of the Interdisciplinary Evidence- and Consensus-based (S3) Guideline “Attention-Deficit/Hyperactivity Disorder (ADHD) in Children, Adolescents and Adults”*. AWMF, 2017 (https://register.awmf.org/de/leitlinien/detail/028-045).

[8] Cortese S, Adamo N, Del Giovane C, Mohr-Jensen C, Hayes AJ, Carucci S, et al. Comparative efficacy and tolerability of medications for attention-deficit hyperactivity disorder in children, adolescents, and adults: a systematic review and network meta-analysis. Lancet Psychiatry 2018; 5(9): 727–38. 30097390 10.1016/S2215-0366(18)30269-4 PMC6109107

[9] Graham J, Coghill D. Adverse effects of pharmacotherapies for attention-deficit hyperactivity disorder: epidemiology, prevention and management. CNS Drugs 2008; 22(3): 213–37. 18278977 10.2165/00023210-200822030-00003

[10] Kooij JJS, Bijlenga D, Salerno L, Jaeschke R, Bitter I, Balázs J, et al. Updated European consensus statement on diagnosis and treatment of adult ADHD. Eur Psychiatry 2019; 56(1): 14–34. 30453134 10.1016/j.eurpsy.2018.11.001

[11] Faraone SV, Rostain AL, Montano CB, Mason O, Antshel KM, Newcorn JH. Systematic review: nonmedical use of prescription stimulants: risk factors, outcomes, and risk reduction strategies. J Am Acad Child Adolesc Psychiatry 2020; 59(1): 100–12. 31326580 10.1016/j.jaac.2019.06.012

[12] Hoxhaj E, Sadohara C, Borel P, D'Amelio R, Sobanski E, Müller H, et al. Mindfulness vs psychoeducation in adult ADHD: a randomized controlled trial. Eur Arch Psychiatry Clin Neurosci 2018; 268(4): 321–35. 29356899 10.1007/s00406-018-0868-4

[13] Vidal R, Bosch R, Nogueira M, Gómez-Barros N, Valero S, Palomar G, et al. Psychoeducation for adults with attention deficit hyperactivity disorder vs. cognitive behavioral group therapy: a randomized controlled pilot study. J Nerv Ment Dis 2013; 201(10): 894–900. 24080677 10.1097/NMD.0b013e3182a5c2c5

[14] Rathbone AL, Prescott J. The use of mobile apps and SMS messaging as physical and mental health interventions: systematic review. J Med Internet Res 2017; 19(8): e295. 28838887 10.2196/jmir.7740 PMC5590007

[15] Iribarren SJ, Cato K, Falzon L, Stone PW. What is the economic evidence for mHealth? a systematic review of economic evaluations of mHealth solutions. PLoS One 2017; 12(2): e0170581. 28152012 10.1371/journal.pone.0170581 PMC5289471

[16] Marcolino MS, Oliveira JAQ, D'Agostino M, Ribeiro AL, Alkmim MBM, Novillo-Ortiz D. The impact of mHealth interventions: systematic review of systematic reviews. JMIR mHealth uHealth 2018; 6(1): e23. 29343463 10.2196/mhealth.8873 PMC5792697

[17] Akbar S, Coiera E, Magrabi F. Safety concerns with consumer-facing mobile health applications and their consequences: a scoping review. J Am Med Inform Assoc 2020; 27(2): 330–40. 31599936 10.1093/jamia/ocz175 PMC7025360

[18] O'Reilly-Jacob M, Mohr P, Ellen M, Petersen C, Sarkisian C, Attipoe S, et al. Digital health & low-value care. Healthc (Amst) 2021; 9(2): 100533. 33714891 10.1016/j.hjdsi.2021.100533 PMC9342901

[19] Lui JHL, Marcus DK, Barry CT. Evidence-based apps? a review of mental health mobile applications in a psychotherapy context. Profess Psychol 2017; 48(3): 199–210.

[20] Larsen ME, Huckvale K, Nicholas J, Torous J, Birrell L, Li E, et al. Using science to sell apps: evaluation of mental health app store quality claims. NPJ Digit Med 2019; 2(1): 18. 31304366 10.1038/s41746-019-0093-1 PMC6550255

[21] Păsărelu CR, Andersson G, Dobrean A. Attention-deficit/ hyperactivity disorder mobile apps: a systematic review. Int J Med Inform 2020; 138: 104133. 32283479 10.1016/j.ijmedinf.2020.104133

[22] Selaskowski B, Steffens M, Schulze M, Lingen M, Aslan B, Rosen H, et al. Smartphone-assisted psychoeducation in adult attention-deficit/hyperactivity disorder: a randomized controlled trial. Psychiatry Res 2022; 317: 114802. 36041353 10.1016/j.psychres.2022.114802

[23] Torous J, Bucci S, Bell IH, Kessing LV, Faurholt-Jepsen M, Whelan P, et al. The growing field of digital psychiatry: current evidence and the future of apps, social media, chatbots, and virtual reality. World Psychiatry 2021; 20(3): 318–35. 34505369 10.1002/wps.20883 PMC8429349

[24] Jang S, Kim J-J, Kim S-J, Hong J, Kim S, Kim E. Mobile app-based chatbot to deliver cognitive behavioral therapy and psychoeducation for adults with attention deficit: a development and feasibility/usability study. Int J Med Inform 2021; 150: 104440. 33799055 10.1016/j.ijmedinf.2021.104440

[25] American Psychiatric Association. Diagnostic and Statistical Manual of Mental Disorders (5th ed.) (5th edn). American Psychiatric Publishing, 2013.

[26] Retz W, Retz-Junginger P, Rösler M. Integrated Diagnosis of ADHD in Adulthood - Revised version (IDA-R). Medice, 2014 (https://www.ida-r-digital.de).

[27] Margraf J, Cwik JC. Mini-DIPS Open Access: Diagnostisches Kurzinterview bei Psychischen Störungen. *[Mini-DIPS Open Access: Diagnostic Short-Interview for Mental Disorders*.*]* Ruhr-Universität Bochum, 2017 (https://www.kli.psy.ruhr-uni-bochum.de/dips-interv/klipsy/download/Mini-DIPS%20Open%20Access.pdf).

[28] Doering S, Renn D, Höfer S, Rumpold G, Smrekar U, Janecke N, et al. Validierung der Deutschen version des fragebogens zur erfassung von DSM-IV persönlichkeitsstorungen (ADP-IV). [Validation of the German version of the questionnaire for the assessment of DSM-IV personality disorders.] Z Psychosom Med Psychother 2007; 53(2): 111–28. 17688782 10.13109/zptm.2007.53.2.111

[29] Faul F, Erdfelder E, Buchner A, Lang A-G. Statistical power analyses using G*power 3.1: tests for correlation and regression analyses. Behav Res Methods 2009; 41(4): 1149–60. 19897823 10.3758/BRM.41.4.1149

[30] Rösler M, Retz W, Retz-Junginger P, Thome J, Supprian T, Nissen T, et al. Instrumente zur diagnostik der aufmerksamkeitsdefizit-/hyperaktivitätsstörung (ADHS) im ErwachsenenalterSelbstbeurteilungsskala (ADHS-SB) und diagnosecheckliste (ADHS-DC). [Tools for the diagnosis of attention-deficit/hyperactivity disorder in adults: self-rating behaviour questionnaire and diagnostic checklist.] Nervenarzt 2004; 75(9): 888–95. 15378249 10.1007/s00115-003-1622-2

[31] THE WHOQOL Group. Development of the World Health Organization WHOQOL-BREF quality of life assessment. Psychol Med 1998; 28(3): 551–8. 9626712 10.1017/s0033291798006667

[32] Nilges P, Essau C. Die depressions-angst-stress-skalen: DASS–ein screeningverfahren nicht nur für schmerzpatienten. [Depression, anxiety and stress scales: DASS—A screening procedure not only for pain patients.] Schmerz 2015; 29(6): 649–57. 26205682 10.1007/s00482-015-0019-z

[33] Merz J, Lehrl S, Galster V, Erzigkeit H. MWT-B - ein intelligenzkurztest. [MWT-B - an intelligence short test.] Psychiat Neurol Med Psychol 1975; 27(7): 423–8. 1197471

[34] D'Amelio R, Retz W, Philipsen A, Rösler M. Psychoedukation und Coaching ADHS im Erwachsenenalter: Manual zur Leitung von Patienten- und Angehörigengruppen (1st edn). [Psychoeducation and Coaching ADHD in Adulthood: Manual for leading patient and family groups.] Elsevier Urban & Fischer, 2009.

[35] Seery C, Wrigley M, O'Riordan F, Kilbride K, Bramham J. What adults with ADHD want to know: a Delphi consensus study on the psychoeducational needs of experts by experience. Health Expect 2022; 25(5): 2593–602. 10.1111/hex.13592 PMC9615057 35999687

[36] Johnson NL. Systems of frequency curves generated by methods of translation. Biometrika 1949; 36(1/2): 149. 18132090

[37] IBM Corp. SPSS Statistics for Windows. IBM Corp, 2012 (https://www.ibm.com/de-de/spss).

[38] R Core Team. R: A Language and Environment for Statistical Computing. R Foundation for Statistical Computing, 2021 (https://www.R-project.org).

[39] Wei T, Simko V, Levy M, Xie Y, Jin Y, Zemla J. R Package 'corrplot'. Statistician 2017; 56(316): e24 (https://cran.r-project.org/web/packages/corrplot/index.html).

[40] Faraone SV, Spencer T, Aleardi M, Pagano C, Biederman J. Meta-analysis of the efficacy of methylphenidate for treating adult attention-deficit/hyperactivity disorder. J Clin Psychopharmacol 2004; 24(1): 24–9. 14709943 10.1097/01.jcp.0000108984.11879.95

[41] Knouse LE, Hu X, Sachs G, Isaacs S. Usability and feasibility of a cognitive-behavioral mobile app for ADHD in adults. PLoS Digit Health 2022; 1(8): 1–19. 10.1371/journal.pdig.0000083 PMC9931323 36812621

