# Supplementary material for: Chatbot-supported psychoeducation in adult attention-deficit hyperactivity disorder: randomised controlled trial
Source: BJPsych Open. 2023 Oct 13;9(6):e192. doi: 10.1192/bjo.2023.573 (PMC10594162; doi:10.1192/bjo.2023.573)
Supplement: Selaskowski et al. supplementary material [file S2056472423005732sup001.pdf]

Supplementary Material

Chatbot-Supported Psychoeducation in Adult Attention-Deficit/Hyperactivity Disorder: Randomized Controlled Trial

Benjamin Selaskowski, Meike Reiland, Marcel Schulze, Behrem Aslan, Kyra Kannen, Annika Wiebe, Torben Wallbaum, Susanne Boll, Silke Lux, Alexandra Philipsen & Niclas Braun

Supplementary Figure 1: Overview of the study design and participant flow from initial contact to final assessment.

Supplementary Figure 2: Group comparisons of correct and missing responses in the psychoeducation content quiz.

Supplementary Table 1: Results of the conducted analyses on primary and secondary outcome parameters.

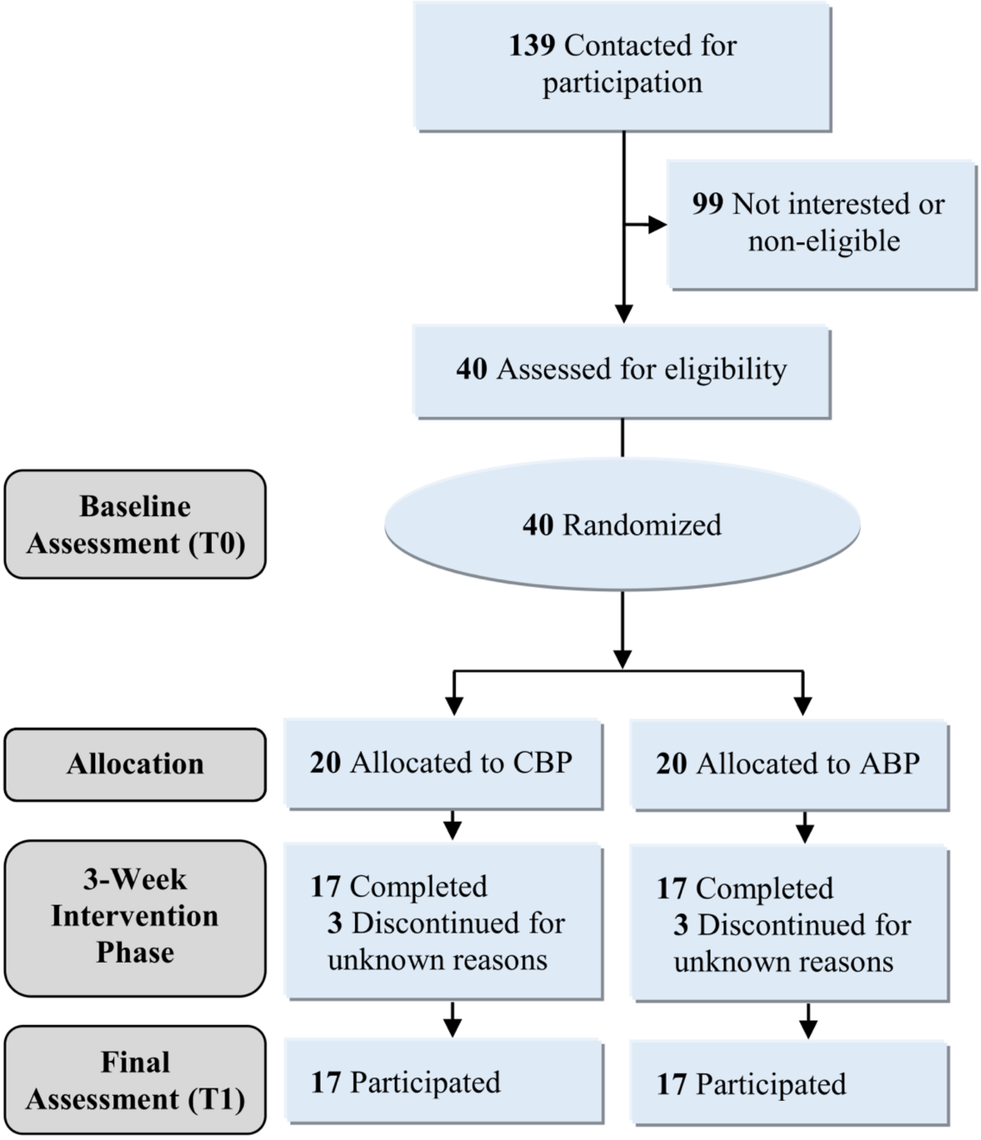

Supplementary Fig. 1. Overview of the study design and participant flow from initial contact to final assessment. A total of 139 individuals were contacted for participation; 40 were comprehensively assessed for study eligibility and all could subsequently be randomized to either chatbot-based psychoeducation (n = 20) or app-based psychoeducation (n = 20). During the course of the interventions, three participants in each group no longer responded to contact attempts and dropped out of the study.

Abbreviations: ABP, app-based psychoeducation; CBP, chatbot-based psychoeducation.

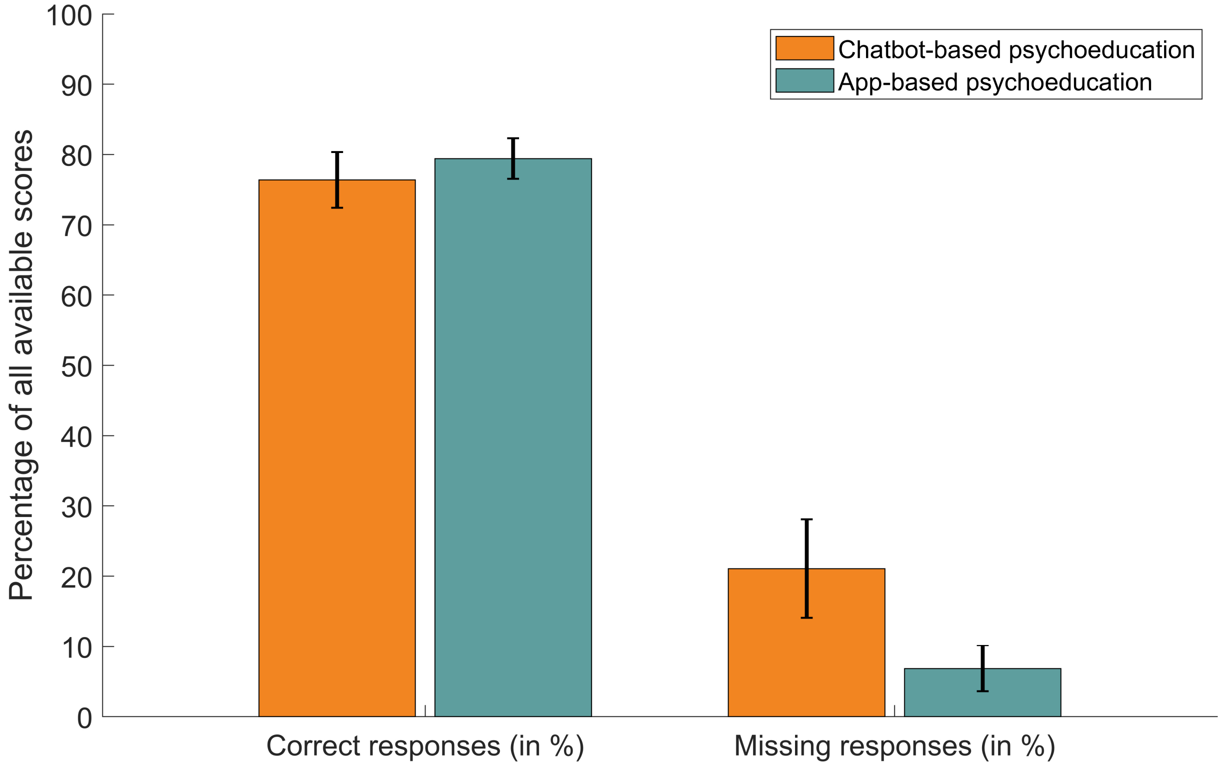

Supplementary Fig. 2. Group comparisons of correct and missing responses in the psychoeducation content quiz. Quizzes were implemented at the end of modules one to seven (four single-choice questions each) to estimate acquired psychoeducation content knowledge. No significant group differences were revealed between the chatbot-based and app-based psychoeducation groups ($p_{correct} = .54$; $p_{missing} = .078$). Error bars indicate standard errors of the mean.

Supplementary Table 1. Results of the conducted analyses on primary and secondary outcome parameters.

Parameter	df	F	*p*	η_p^2
IDA-R (observer-rated ADHD symptoms)				
Total Time	1,32	44.44	< .001	.58
Total Group	1,32	3.47	.072	.098
Total Time × Group Interaction	1,32	0.04	.84	.001
Inattention Time	1,32	30.30	< .001	.47
Inattention Group	1,32	3.21	.083	.091
Inattention Time × Group Interaction	1,32	0.17	.68	.005
Hyperactivity Time	1,32	18.30	< .001	.36
Hyperactivity Group	1,32	0.72	.40	.02
Hyperactivity Time × Group Interaction	1,32	0.16	.69	.005
Impulsivity Time	1,32	34.90	< .001	.52
Impulsivity Group	1,32	2.10	.16	.061
Impulsivity Time × Group Interaction	1,32	0.15	.70	.005
ADHS-SB (self-rated ADHD symptoms)				
Total Time	1,32	7.12	.012	.18
Total Group	1,32	1.93	.17	.057
Total Time × Group Interaction	1,32	0.03	.88	.001
Inattention Time	1,32	6.77	.014	.18
Inattention Group	1,32	0.16	.69	.005
Inattention Time × Group Interaction	1,32	1.17	.29	.035
Hyperactivity Time	1,32	7.64	.009	.19
Hyperactivity Group	1,32	1.73	.20	.051
Hyperactivity Time × Group Interaction	1,32	< 0.01	.96	< .001
Impulsivity Time	1,32	1.65	.21	.049
Impulsivity Group	1,32	4.84	.035	.13
Impulsivity Time × Group Interaction	1,32	1.65	.21	.049
DASS (depression, anxiety and stress symptoms)				

Depression Time	1,32	< 0.01	.97	< .001
Depression Group	1,32	1.18	.29	.035
Depression Time × Group Interaction	1,32	2.47	.13	.072
Anxiety Time	1,32	0.12	.73	.004
Anxiety Group	1,32	1.70	.20	.05
Anxiety Time × Group Interaction	1,32	0.72	.40	.022
Stress Time	1,32	1.51	.23	.045
Stress Group	1,32	5.79	.022	.15
Stress Time × Group Interaction	1,32	0.01	.94	< .001
WHOQoL (WHO quality of life)				
Physical health Time	1,32	0.17	.69	.005
Physical health Group	1,32	0.16	.69	.005
Physical health Time × Group Interaction	1,32	< 0.01	.95	< .001
Psychological health Time	1,32	1.39	.25	.042
Psychological health Group	1,32	.007	.94	< .001
Psychological health Time × Group Interaction	1,32	0.47	.50	.014
Social relationships Time	1,32	1.64	.21	.049
Social relationships Group	1,32	4.27	.047	.12
Social relationships Time × Group Interaction	1,32	0.36	.55	.011
Environment Time	1,32	1.77	.19	.052
Environment Group	1,32	1.53	.23	.046
Environment Time × Group Interaction	1,32	0.12	.73	.004
**PE knowledge quiz**				
Missing	t(22,5)	-1.85	.078	d = -.078
Proportion correct	t(32)	0.62	.54	d = 0.22
